# A Commensal Streptococcus Dysregulates the *Pseudomonas aeruginosa* Nitrosative Stress Response

**DOI:** 10.3389/fcimb.2022.817336

**Published:** 2022-05-10

**Authors:** Joshua J. Baty, Joshua T. Huffines, Sara N. Stoner, Jessica A. Scoffield

**Affiliations:** Department of Microbiology, School of Medicine, University of Alabama at Birmingham, Birmingham, AL, United States

**Keywords:** nitrosative stress, *Pseudomonas aeruginosa*, *Streptococcus parasanguinis*, polymicrobial, denitrification

## Abstract

Chronic infections in the cystic fibrosis (CF) airway are composed of both pathogenic and commensal bacteria. However, chronic *Pseudomonas aeruginosa* infections are the leading cause of lung deterioration in individuals with CF. Interestingly, oral commensals can translocate to the CF lung and their presence is associated with improved lung function, presumably due to their ability to antagonize *P. aeruginosa*. We have previously shown that one commensal, *Streptococcus parasanguinis*, produces hydrogen peroxide that reacts with nitrite to generate reactive nitrogen intermediates (RNI) which inhibit *P. aeruginosa* growth. In this study, we sought to understand the global impact of commensal-mediated RNI on the *P. aeruginosa* transcriptome. RNA sequencing analysis revealed that *S. parasanguinis* and nitrite-mediated RNI dysregulated expression of denitrification genes in a CF isolate of *P. aeruginosa* compared to when this isolate was only exposed to *S. parasanguinis*. Further, loss of a nitric oxide reductase subunit (*norB*) rendered an acute *P. aeruginosa* isolate more susceptible to *S. parasanguinis-*mediated RNI. Additionally, *S. parasanguinis*-mediated RNI inactivated *P. aeruginosa* aconitase activity. Lastly, we report that *P. aeruginosa* isolates recovered from CF individuals are uniquely hypersensitive to *S. parasanguinis*-mediated RNI compared to acute infection or environmental *P. aeruginosa* isolates. These findings illustrate that *S. parasanguinis* hinders the ability of *P. aeruginosa* to respond to RNI, which potentially prevents *P. aeruginosa* CF isolates from resisting commensal and host-induced RNI in the CF airway.

## Introduction

Oral commensal bacteria play a variety of roles in the oral cavity, such as maintaining health and homeostasis, metabolizing host-derived nutrients, and synthesizing antimicrobials such as hydrogen peroxide (H_2_O_2_) to antagonize and outcompete oral pathogens (Doel et al., 2005; Kreth et al., 2008; Schreiber et al., 2010; Hanel et al., 2020). Interestingly, oral commensal bacteria have also been shown to have systemic effects, most notably, modulating blood pressure through the generation of nitric oxide and interacting with chronic airway pathogens (Scoffield and Wu, 2015; Flynn et al., 2016; Bryan et al., 2017; Scoffield et al., 2017; Joshipura et al., 2020). Polymicrobial competition between oral commensals and pathogens are particularly observed in cystic fibrosis (CF) airway disease, where these commensals have been found to be associated with improved lung function and clinical stability (Filkins et al., 2012; Cuthbertson et al., 2020). CF is the result of a mutated cystic fibrosis transmembrane conductor regulator channel (CFTR) through which anions are not transported across the plasma membrane, leading to a dehydrated periciliary layer in the airway (Derichs et al., 2011). Loss of proper anion transport results in the accumulation of thick, sticky mucus that allows for a variety of microbes to colonize (Palmer et al., 2007; Filkins et al., 2012; Cowley et al., 2015; Flynn et al., 2016; Hogan et al., 2016; Cuthbertson et al., 2020).

One of the most common and clinically important CF pathogens is the opportunistic pathogen *Pseudomonas aeruginosa.* As a human pathogen that can survive in diverse environments, *P. aeruginosa* is quite adaptable to a wide variety of ecosystems ranging from soil and aquatic ecosystems to the human lung (Favero et al., 1971; Green et al., 1974; Khan et al., 2007; Filkins et al., 2012; Crone et al., 2020). As such, one pathway that serves *P. aeruginosa* in a variety of niches, including the CF lung, is the denitrification pathway. Nitrate and its derivatives can act as final electron acceptors when oxygen is limited (Carlson and Ingraham, 1983; Schreiber et al., 2007; Borrero-de Acuña et al., 2016). Nitrate and its derivatives are found in the CF lung, which contains many anoxic pockets for *P. aeruginosa* to grow anaerobically through denitrification (Grasemann et al., 1998; Ho et al., 1998; Linnane et al., 1998; Balint et al., 2001; Olin et al., 2001; Line et al., 2014; Cowley et al., 2015). Remarkably, several genes in the denitrification pathway have been directly linked to virulence factor production and pathogenesis (Filiatrault et al., 2006; Van Alst et al., 2007). Due to its arsenal of virulence factors, large and adaptable genome, and its intrinsic drug resistance, *P. aeruginosa* is a major contributor of morbidity and mortality in the CF population (Emerson et al., 2002; Winstanley et al., 2016; Pang et al., 2019; Patient-Registry-Annual-Data-Report.Pdf, 2020).

While the contribution of major pathogens like *P. aeruginosa* in CF lung disease is well known, the role of oral commensal bacteria that have been found to reside in the CF lung are largely unknown. Previous studies from our lab have shown that the oral bacterium *Streptococcus parasanguinis*, a commensal associated with improved lung function in CF, can inhibit *P. aeruginosa* in a nitrite-dependent manner, and perhaps contribute to improved lung function in individuals who are colonized with *S. parasanguinis* (Filkins et al., 2012; Scoffield and Wu, 2015; Cuthbertson et al., 2020). H_2_O_2_ produced by *S. parasanguinis* can react with nitrite to form peroxynitrite and other reactive nitrogen intermediates (RNI) that are lethal to *P. aeruginosa* (Scoffield and Wu, 2015). Nitrite and its derivatives are found in the CF lung, and the presence of NO_x_ have been associated with improved lung function (Grasemann et al., 1998; Ho et al., 1998; Balint et al., 2001). However, *P. aeruginosa* can perform denitrification and is capable of reducing nitrate and its derivatives to nitrogen, indicating that *P. aeruginosa* is at least partially capable of tolerating RNI (Borrero-de Acuña et al., 2016). We previously reported that nitrite reductase (*nirS*), an enzyme in the denitrification pathway, is essential for *P. aeruginosa* to withstand *S. parasanguinis*-mediated nitrosative stress. The expression of *nirS* is reduced in a lab-adapted chronic CF isolate and this isolate displays increased susceptibility to *S. parasanguinis*-mediated RNI compared to a lab-adapted acute isolate *in vitro* (Scoffield and Wu, 2016), suggesting that chronic CF isolates may have acquired global alterations in genes that are required for them to respond to and resist RNI.

Despite differential responses to commensal mediated-RNI between chronic and acute *P. aeruginosa* isolates, the global impact of *S. parasanguinis* on the *P. aeruginosa* RNI-responsive transcriptome is unclear. To better understand how *S. parasanguinis* and nitrite modulate the *P. aeruginosa* nitrosative stress response, we utilized RNA sequencing to determine how commensal-derived RNI modulate the *P. aeruginosa* RNI-responsive transcriptome in both an acute non-CF and a chronic CF isolate of *P. aeruginosa*. In this study, we report that *S. parasanguinis* reduces the expression of several denitrification genes in the CF isolate compared to the acute isolate. However, the expression of nitric oxide reductase (*norCB*) was similarly upregulated in both the chronic and acute isolates, indicating that nitric oxide reductase is an essential component for *P. aeruginosa* tolerance to RNI. Due to this result, we hypothesized that loss of *norCB* may trigger hypersensitivity to RNI in an acute isolate. Loss of *norC* and *norB* resulted in hypersensitivity to *S. parasanguinis*-mediated RNI in the acute isolate. Further, *S. parasanguinis* alone and with nitrite significantly decreased aconitase (an enzyme readily inactivated by RNI) activity in the acute background in the *norB* mutant, indicating that *norB* is critical for the *P. aeruginosa* nitrosative stress response. Finally, we demonstrate that clinical CF isolates are uniquely sensitive to *S. parasanguinis* and nitrite-mediated RNI compared to acute or environmental isolates. Taken together, this work illustrates that chronic CF isolates of *P. aeruginosa* have altered nitrosative stress responses that render them vulnerable to RNI. Lastly, due to increased susceptibility to RNI in the nitric oxide reductase mutants, *norCB* could potentially be targeted as a therapeutic strategy to treat recalcitrant *P. aeruginosa* infections.

## Materials and Methods

### Bacterial Strains, Culture Conditions, and Reagents

The bacteria used in this study were *S. parasanguinis* (FW213), *P. aeruginosa* (PAO1, FRD1, clinical isolates from CF and non-CF individuals, and environmental isolates), and Escherichia coli (DH10b) (**Table 1**). *P. aeruginosa* was maintained on *Pseudomonas* isolation agar (PIA: 20g peptone, 1.4g magnesium chloride, 10g potassium sulfate, 25mg Irgasan, 21.1g agar, 12.5g LB per liter) and subsequently cultured in Lysogeny broth (LB: 10g tryptone, 5g yeast extract, 10g sodium chloride per liter) and incubated at 37°C shaking at 250rpm. *E. coli* cells were also cultured in LB and incubated at 37°C shaking at 250rpm. *S. parasanguinis* was cultured in Todd-Hewitt broth (THB: 3.1g heart infusion, 20g neopeptone, 2g dextrose, 2g sodium chloride, 0.4g disodium phosphate, 2.5g sodium carbonate per liter) and incubated at 37°C with 5% CO_2_. Filter sterilized sodium nitrite (Waltham, MA, USA) was used at a concentration of 0.5 or 1mM where indicated. Antibiotics were purchased from Sigma-Aldrich (St. Louis, MO, USA) and used at the following concentrations: 100µg of ampicillin ml^-1^ for *E. coli*; 40µg of erythromycin ml^-1^ for *E. coli*; and 180 µg of gentamicin ml^–1^ and 100 µg of carbenicillin ml^–1^ for *P. aeruginosa*.

**Table 1:** Bacterial strains and plasmids used in this study.

Strain	Characteristics	Reference/Source
FRD1 (*P. aeruginosa*)	Lab adapted isolate from a CF individual, mucoid	(Ohman and Chakrabarty, 1981)
PAO1 (*P. aeruginosa*)	Lab adapted wound isolate, non-mucoid	(Holloway et al., 1979)
PAO1 *norB* (*P. aeruginosa*)	In-frame deletion of *norB*	(Jacobs et al., 2003; Held et al., 2012)
PAO1 *norC* (*P. aeruginosa*)	In-frame deletion of *norC*	(Jacobs et al., 2003; Held et al., 2012)
PAO1 *norB+* (*P. aeruginosa*)	Complemented PAO1 *norB* mutant	This study
PAO1 *norC+* (*P. aeruginosa*)	Complemented PAO1 *norC* mutant	This study
Environmental isolates (*P. aeruginosa*)	E1-E10 non-mucoid, water isolates	This study
AC1 (*P. aeruginosa*)	Non-mucoid, urine isolate	Dr. Bill Benjamin UAB Clinical Microbiology Lab
AC2 (*P. aeruginosa*)	Non-mucoid, wound isolate	Dr. Bill Benjamin UAB Clinical Microbiology Lab
AC3 (*P. aeruginosa*)	Non-mucoid, urine isolate	Dr. Bill Benjamin UAB Clinical Microbiology Lab
AC4 (*P. aeruginosa*)	Non-mucoid, bronchial wash isolate	Dr. Bill Benjamin UAB Clinical Microbiology Lab
AC5 (*P. aeruginosa*)	Non-mucoid, urine isolate	Dr. Bill Benjamin UAB Clinical Microbiology Lab
AC6 (*P. aeruginosa*)	Non-mucoid, blood isolate	Dr. Bill Benjamin UAB Clinical Microbiology Lab
AC7 (*P. aeruginosa*)	Non-mucoid	Dr. Bill Benjamin UAB Clinical Microbiology Lab
AC8 (*P. aeruginosa*)	Non-mucoid, bronchial wash isolate	Dr. Bill Benjamin UAB Clinical Microbiology Lab
AC9 (*P. aeruginosa*)	Non-mucoid, urine isolate	Dr. Bill Benjamin UAB Clinical Microbiology Lab
AC10 (*P. aeruginosa*)	Non-mucoid, trachael wash isolate	Dr. Bill Benjamin UAB Clinical Microbiology Lab
AC11 (*P. aeruginosa*)	Non-mucoid, urine isolate	Dr. Bill Benjamin UAB Clinical Microbiology Lab
AC12 (*P. aeruginosa*)	Non-mucoid, urine isolate	Dr. Bill Benjamin UAB Clinical Microbiology Lab
AC13 (*P. aeruginosa*)	Sinus isolate	Dr. Bill Benjamin UAB Clinical Microbiology Lab
CF clinical isolates (*P. aeruginosa*)	CF1-CF7 Isolates 1,3,5, and 7, mucoid, and 2, 4, 6 non-mucoid	Dr. Susan Birket UAB CF Center
*S. parasanguinis* FW213	Wild type	(Cole et al. 1976)
*S. parasanguinis poxL*	H_2_O_2_ deficient mutant	(Scoffield and Wu, 2015)
*S. parasanguinis poxL^+^*	Complemented for *poxL*	(Scoffield and Wu, 2015)
DH10b (*E. coli*)	Host strain for cloning	Thermo Fisher
pJB1	pBluescript K(+) plasmid ligated to *norB*	This study
pJB2	pBluescript K(+) plasmid ligated to *norC*	This study

### RNA Sequencing

*P. aeruginosa* strains PAO1 or FRD1 were grown in the presence or absence of *S. parasanguinis* FW213 with or without 0.5mM nitrite, a sub-inhibitory concentration of nitrite. Cultures were grown in three biological replicates with 50µL overnight inoculum in 10mL Todd Hewitt Broth (THB) at 37°C with 5% CO_2_. Cultures were harvested at an absorbance of 600nm at 0.72-0.78. RNA was purified using the Zymo Research Direct-zol RNA Miniprep according to the manufacturer’s directions (Cat: R2052) (Irvine, CA, USA). mRNA sequencing was performed at the UAB Heflin Center for Genomic Sciences using an Illumina NextSeq 500 as described by the manufacturer (Illumina, San Diego, CA, USA). Use of an Agilent SureSelect strand-specific mRNA library kit (Agilent, Santa Clara, CA, USA) and ribosome reduction with the RiboMinus protocol for Gram-negative and Gram-positive bacteria (Life Technologies, Carlsbad, CA, USA) were performed as described by the manufacturers. The resulting mRNA was randomly fragmented with cations and heat, followed by first-strand synthesis using random primers. Second-strand cDNA production was done with standard techniques, and cDNA libraries were quantified using qPCR in a Roche LightCycler 480 (Roche, Basel, Switzerland) with a Kapa Biosystems kit for Illumina library quantitation prior to cluster generation, which was performed according to the manufacturers’ recommendations for onboard clustering (Illumina). Approximately 12 million double-stranded 50-bp reads were generated per sample. Data were analyzed using DESeq2. Species-specific genes analyzed with HTSeq that had the highest *P* values are reported. RNA sequencing data have been deposited in NCBI’s Gene Expression Omnibus and are accessible through GEO series accession number PRJNA779943.

### Construction of the *P. aeruginosa norB, norC*, and Complemented Strains

The *norB* and *norC* mutants were obtained from the University of Washington *P. aeruginosa* transposon mutant library (University of Washington, Seattle, WA, USA). To complement the *norB* and *norC* mutants, the full-length coding region from each gene was PCR-purified from PAO1 cells and cloned into the EcoR1 and Xba1, and EcoR1 and BamH1 sites of pBluescript K(+), respectively. The primers used to amplify *norB* and *norC* were forward- 5’-ATGATGTCGCCCAATGGCCTCC-3’/reverse- 5’-TCAGGCGGCCGCCTT-3’ and forward- 5’-CATCAGAATTCGTCGGGAGTGCTCTACTGGC-3’/reverse-5’CATCAGGATCCTCAACCCTCCTTGTTCGGCG-3’, respectively. The resulting plasmids (pJB1 for *norB* and pJB2 for *norC*) were converted to a mobilized plasmid *via* the addition of a m*oriT* at the HindIII site and then introduced into the *norB* and *norC* mutants through triparental mating.

### Competition Assays & Quantification of Zones of Inhibition

10µL of overnight *S. parasanguinis*, the *S. parasanguinis poxL* mutant, or *S. parasanguinis poxL^+^* complemented strain was spotted onto LB agar plates with or without 1mM nitrite. Plates were grown for 18 hours at 37°C at 5% CO_2_. The next day, *P. aeruginosa* (either PAO1, FRD1, *norB, norC, norB+, norC+*, or clinical CF, clinical acute, or environmental isolates) was subcultured to an OD of 0.1 at an absorbance of 600nm and 10µL was spotted next to *S. parsanguinis*. Plates were grown overnight at 30°C at 5% CO_2_. Inhibition of *P. aeruginosa* growth was assessed by the presence or absence of zones of inhibition by *S. parasanguinis.* Zones of inhibition for three biological replicates with two technical replicates were quantified using ImageJ software (Abramoff et al., 2004; Schneider et al., 2012).

### Aconitase Assays and CFU Enumeration

Single species cultures of *P. aeruginosa* were grown in 12 well Costar microtiter plates (Corning, Inc., Corning, NY, USA). Dual cultures of *P. aeruginosa* and *S. parasanguinis* were grown in 12 well transwell plates with a 0.4µm polyester membrane insert. *P. aeruginosa* was grown basally with *S. parasanguinis* grown apically. (Corning, Inc., Corning, NY, USA). *S. parasanguinis* (50µL) and *P. aeruginosa* (150µL) were inoculated simultaneously and were grown for 5 hours together. Aconitase activity was measured using the EnzyChrom Aconitase Assay Kit (BioAssay Systems, Hayward, CA, USA). CFU were enumerated through serial dilution and spot plating on LB agar. Three biological replicates were utilized for aconitase assays.

### Statistical Analysis

All statistics were performed using Graphpad Prism (Graphpad Prism version 9.2.0 for Windows Graphpad Software, San Diego, California, USA). Normality was assessed using the Shapiro-Wilk test. Further statistical tests were performed where indicated.

## Results

### *S. parasanguinis* Alters the Expression of *P. aeruginosa* Denitrification Pathway

**Figure 1:**
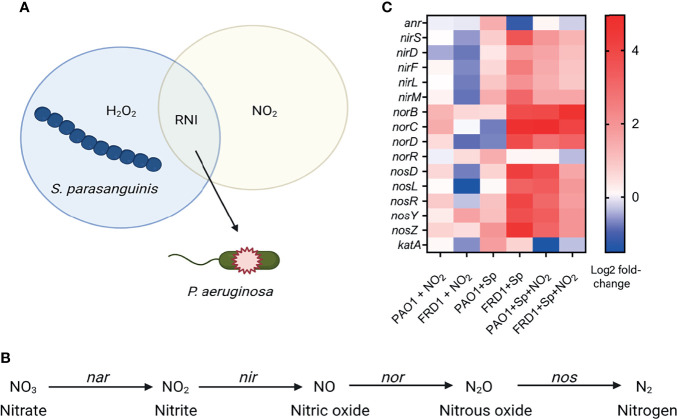
*S. parasanguinis* dysregulates the *P. aeruginosa* nitrate reduction transcriptome. **(A)** Oral commensal Streptococci such as *S. parasanguinis* produce hydrogen peroxide that can react with endogenous nitrite to form reactive nitrogen intermediates (RNI) such as peroxynitrite that are lethal to *P. aeruginosa.* **(B)** *P. aeruginosa* denitrification pathway. Nitrate is reduced by nitrate reductase (Nar), nitrite is reduced by nitrite reductase (Nir), nitric oxide is reduced by nitric oxide reductase (Nor), nitrous oxide is reduced by nitrous oxide reductase (Nos). **(C)** Heat map generated from RNA sequencing showing the relative transcription of genes involved in denitrification between PAO1 and FRD1 in the presence of nitrite, *S. parsanguinis*, and nitrite and *S. parsanguinis*.

We previously reported that H_2_O_2_ production by *S. parasanguinis*, which requires pyruvate oxidase (*poxL*), and nitrite inhibit *P. aeruginosa via* the production of RNI (**Figure 1A**) (Scoffield and Wu, 2015; Scoffield and Wu, 2016). Although *P. aeruginosa* possesses a denitrification pathway that contains a group of reductase enzymes and genes (nitrate reductase/*nar*, nitrite reductase/*nir*, nitric oxide reductase/*nor*, and nitrous oxide reductase/*nos*) that can reduce nitrate to nitrogen (**Figure 1B**), and thereby detoxify certain RNI, our previous observation showing hypersensitivity of a chronic CF isolate to commensal-mediated RNI prompted us to question whether *S. parasanguinis* impedes the ability of chronic *P. aeruginosa* to sufficiently respond to RNI. To determine the effects of *S. parasanguinis* and nitrite on the *P. aeruginosa* denitrification transcriptome, RNA sequencing was performed on two different isolates of *P. aeruginosa*— a lab adapted acute wound isolate, PAO1, or a lab adapted chronic CF isolate, FRD1— in the presence or absence of either 0.5mM nitrite, *S. parasanguinis*, or the combination of nitrite and *S. parasanguinis.* **Figure 1C** shows the relative gene expression of the relevant reductase genes along with the anaerobic nitrate reduction regulator gene (*anr*) and the catalase gene (*katA*) that is responsive to H_2_O_2_. In the presence of nitrite, PAO1 upregulated expression of the majority of reductase genes (*norB*, *norC*, *norD*, *nosD*, *nosR*, and *nosZ*) required to reduce nitrite to nitrogen while FRD1 downregulated expression of these relevant genes, except for *nosY* (**Figure 1C**). Neither *anr* nor *katA* were upregulated in the presence of nitrite in PAO1 or FRD1. In contrast to nitrite exposure, the presence of *S. parasangunis* alone upregulated denitrification gene expression in both PAO1 and FRD1, but to a higher degree in FRD1. The expression of *katA* was upregulated in both PAO1 and FRD1 in the presence of *S. parasanguinis*, while *anr* was upregulated in PAO1 and downregulated in FRD1. When *S. parasanguinis* and nitrite were both present, which generates RNI (Scoffield and Wu, 2015), PAO1 showed a small increase in *anr* expression while FRD1 downregulated *anr.* Additionally, the expression of *katA* was downregulated in both PAO1 and FRD1. PAO1 increased the expression of all denitrification genes, particularly the nitric oxide reductase subunits B and C (**Figure 1C**). Conversely, FRD1 was less responsive to *S. parasanguinis* and nitrite-mediated RNI compared to PAO1, indicating that the chronic CF isolate FRD1 may be particularly susceptible to nitrosative stress. Interestingly, the FRD1 isolate expressed nitric oxide reductase B or C subunits to a similar degree as PAO1 in the presence of *S. parasanguinis* and nitrite, indicating that chronic *P. aeruginosa* relies particularly on nitric oxide reductase to tolerate RNI, even when other enzymes in the denitrification complex are downregulated compared to the acute isolate. Thus, we hypothesized that nitric oxide reductase is essential for *P. aeruginosa* RNI tolerance. As the NorD subunit of nitric oxide reductase has not been shown to have enzymatic activity (Bartnikas et al., 1997; Yamagiwa et al., 2018), we chose to pursue the roles of the NorB and NorC subunits in the *P. aeruginosa* nitrosative stress response.

### *norB* Is Essential for *P. aeruginosa* Nitrosative Stress Tolerance

**Figure 2:**
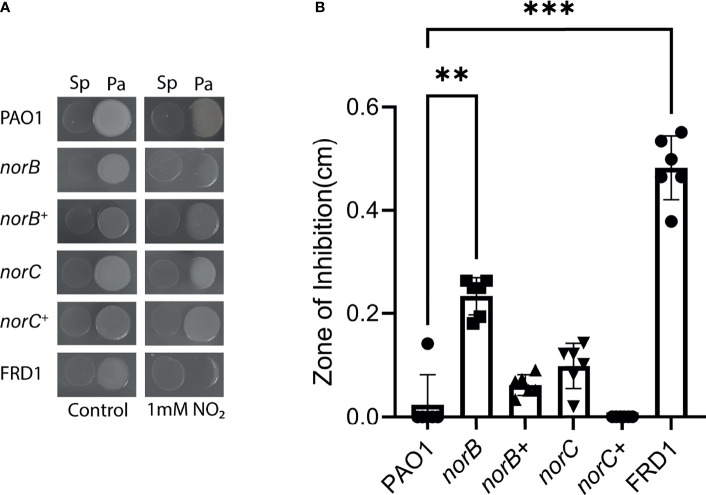
*norB* and *norC* are required for resistance to reactive nitrosative intermediates. **(A)** *P. aeruginosa* PAO1*, norB* mutant*, norB^+^* complemented*, norC* mutant, and *norC^+^* complemented, and FRD1 strains were spotted next to *S. parasanguinis* in the presence or absence of 1mM nitrite. **(B)** Zones of inhibition in the presence of *S. parasanguinis* and nitrite were quantified for PAO1, *norB* mutant*, norB^+^* complemented*, norC* mutant*, norC^+^* complemented, and FRD1 strains. n = 6, 3 biological replicates with 2 technical replicates, error bars represent standard deviation, **P < 0.01, ***P < 0.001 (Kruskal-Wallis, Dunnett *Post Hoc* Test).

Due to similar expression of *norB* and *norC* between PAO1 and FRD1 in the presence of both *S. parasanguinis* and nitrite, we hypothesized that *norB* and *norC* are necessary for *P. aeruginosa* to tolerate nitrosative stress. Based on our previous finding that FRD1 is exceptionally sensitive to *S. parasanguinis* and nitrite compared to PAO1, we reasoned that loss of *norB* and *norC* in the FRD1 background would result in complete lethality (Scoffield and Wu, 2016). Therefore, *norB* and *norC* deletion mutants were tested in the PAO1 background for enhanced sensitivity to *S. parasanguinis-*induced nitrosative stress using a plate competition assay. While both the *norB* and the *norC* mutants displayed enhanced sensitivity to *S. parasanguinis* and nitrite-mediated RNI, the *norB* mutant had markedly reduced growth compared to PAO1 (**Figures 2A, B**), indicating that the *norB* subunit of nitric oxide reductase is more functionally necessary for RNI tolerance. Enhanced inhibition of *norB* and *norC* by *S. parasanguinis* and nitrite was restored by *norB* and *norC* complementation (**Figures 2A, B**). Further, in agreement with our previously reported findings, loss of *poxL*, which encodes for pyruvate oxidase and is required for H_2_O_2_ production, abolished nitrite-dependent inhibition of *P. aeruginosa* by *S. parasanguinis*; however, inhibition was restored by the *poxL* ^+^ complemented strain (**Figures S1A–G**). It is important to note that *S. parasanguinis* grows more translucently on agar plates. Additionally, the growth of this commensal is not affected in the presence of *P. aeruginosa* or when grown alone on agar plates with or without nitrite (**Figure S1A**). The growth of all *P. aeruginosa* strains was also unaffected in the single controls on agar plates with or without nitrite (**Figure S1**); however, planktonic growth was slightly reduced in FRD1 and the PAO1 *norB* mutant in the presence of nitrite alone (**Figure S2**).

**Figure 3:**
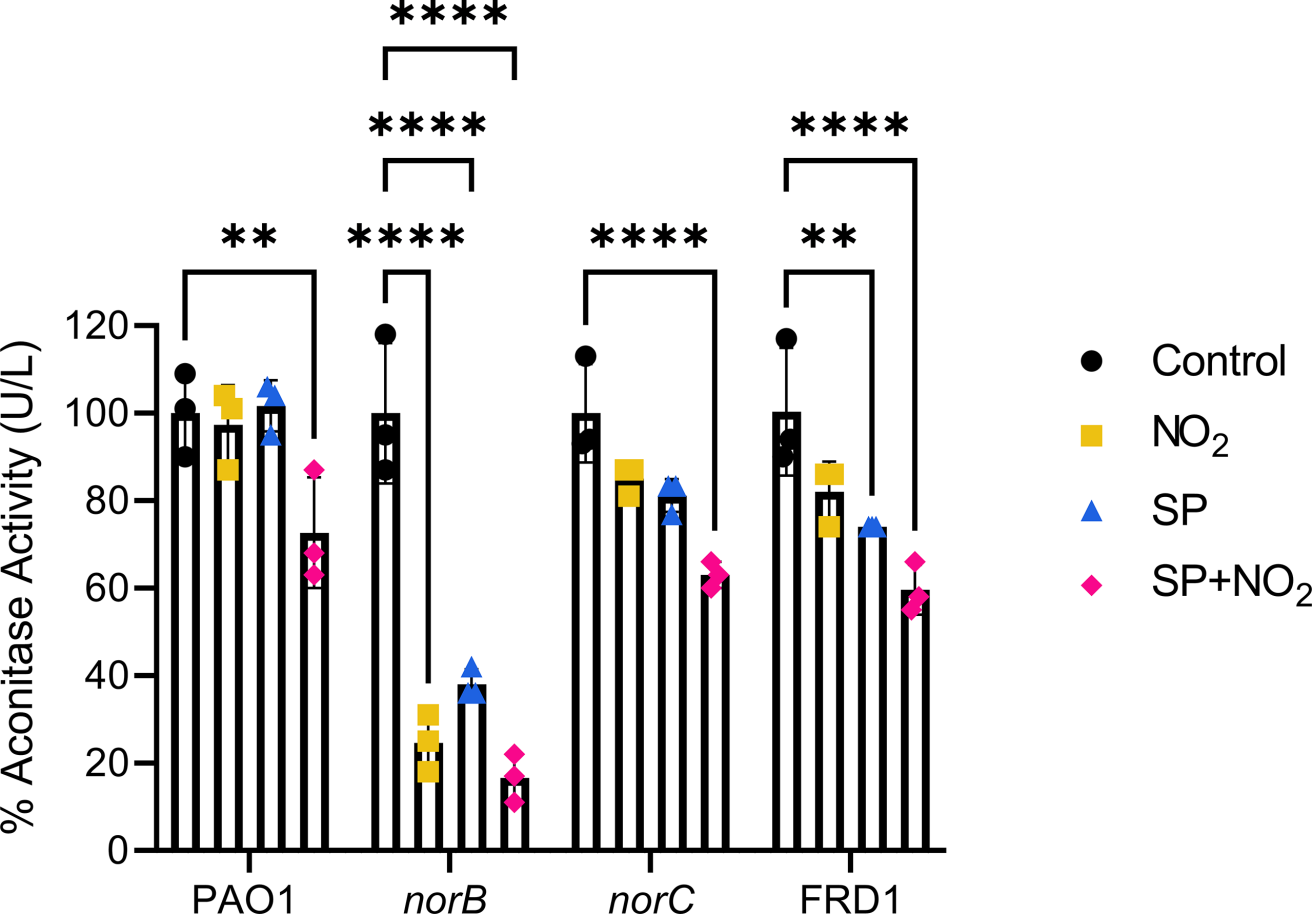
*S. parsanguinis* and nitrite reduce aconitase activity in the norB mutant. PAO1, *norB, norC*, and FRD1 were exposed to nitrite, *S. parasanguinis*, and nitrite and *S. parsanguinis*. Aconitase activity was measured and normalized to the average of the controls. n=3, error bars represent standard deviation, **P < 0.01, ****P < 0.0001 (Two-Way ANOVA, Tukey *Post Hoc* Test).

Next, to determine the direct effects of *S. parasanguinis*-mediated nitrosative stress on *P. aeruginosa*, we assessed aconitase activity. Aconitase, an enzyme in the tricarboxylic acid cycle (TCA) that catalyzes the conversion of citrate to isocitrate, has an iron-sulfur cluster in its active site that is prone to oxidation and nitrosylation (Gardner et al., 1997; Su et al., 2014). Therefore, any nitrosylation event from RNIs will decrease aconitase activity. Aconitase activity in PAO1, *norB, norC*, and FRD1 was assessed in the presence of nitrite, *S. parasanguinis*, and the combination of nitrite and *S. parasagnuinis*. Dual species cultures were grown in transwell plates where *S. parasanguinis* was incubated in the insert above *P. aeruginosa* in which the two species were not in physical contact with one another. The combination of both *S. parasanguinis* and nitrite decreased aconitase activity to differing degrees in all isolates without reducing cell number in both PAO1 and FRD1, indicating that reduced aconitase activity is not due to cell death (**Figures 3** and **S3**). The *norB* mutant was especially sensitive to *S. parasanguinis-*mediated RNI where activity was reduced by more than 50%. Additionally, the *norB* mutant had reduced aconitase activity in the presence of nitrite alone, indicating that impaired reduction of nitric oxide leads to its accumulation and activity on aconitase. FRD1 and *norB* also had reduced aconitase activity in the presence of *S. parasanguinis* alone, presumably due to oxidative stress from *S. parasanguinis-*generated H_2_O_2_. To determine the effect of H_2_O_2_ produced by *S. parasanguinis* on *P. aeruginosa* aconitase activity, a *S. parasanguinis* strain defective for H_2_O_2_ production (*poxL* mutant) was employed. Aconitase activity was fully restored in both the *norB* and *norC* mutants in the presence of the *S. parasanguinis poxL* mutant (**Figure S4**). In the presence of the *S. parasanguinis poxL* mutant and nitrite, *norB* aconitase activity was comparable to the presence of nitrite alone while *norC* aconitase activity was restored. Interestingly, the presence of the *poxL* mutant did not increase aconitase activity in FRD1. While the deletion of *poxL* largely reduces H_2_O_2_ production, some residual H_2_O_2_ is produced. Given that FRD1 is hypersensitive to both oxidative and nitrosative stress, minute concentrations of H_2_O_2_ may be enough to inhibit its aconitase activity (Scoffield and Wu, 2015; Ge et al., 2016).

### Chronic CF Isolates Are Hypersensitive to *S. parasanguinis*-Mediated RNI

**Figure 4:**
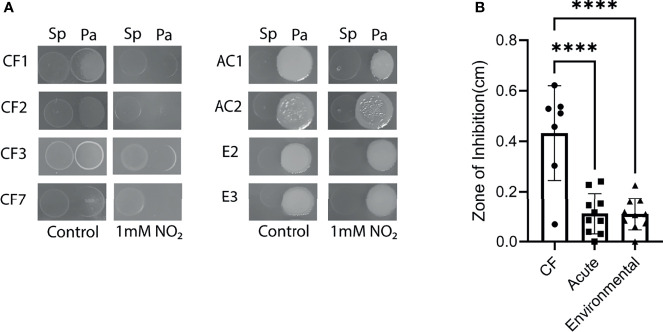
Clinical CF isolates of *P. aeruginosa* are sensitive to *S. parasanguinis-*induced nitrosative stress. **(A)** Representative clinical, acute, and environmental isolates of *P. aeruginosa* were spotted next to *S. parasanguinis* in the presence or absence of 1mM nitrite. **(B)** Average zone of inhibition for each isolate was calculated. Isolates were grouped into CF isolates, acute isolates, or environmental isolates. Zones of inhibition of each group were compared. CF, n=7; Acute, n=10; Environmental, n=10. Error bars represent standard deviation, ****P < 0.0001 (Kruskal-Wallis, Dunnett *Post Hoc* Test).

Given the clear distinctions between PAO1 and FRD1 tolerance to RNI, we hypothesized that other chronic CF isolates would be uniquely sensitive to RNI compared to acute non-CF and environmental isolates of *P. aeruginosa.* To test this hypothesis, competition assays were performed where *P. aeruginosa* isolates were spotted next to *S. parasanguinis* in the presence or absence of nitrite. Interestingly, all of the chronic CF *P. aeruginosa* isolates were sensitive to *S. parasanguinis*-mediated RNI (**Figures 4** and **S5**), and isolates CF2 and CF7 displayed some sensitivity to *S. parasanguinis* without nitrite which was attributed to H_2_O_2_ production (*poxL*). Loss of *poxL* abolished nitrite-dependent inhibition in all chronic isolates and complementation of *poxL* restored inhibition (**Figures S5A–H**). Acute and environmental isolates were less sensitive to *S. parasanguinis-*mediated RNI than the CF isolates, with several isolates demonstrating little to no inhibition (**Figures S6A–K** and **S7A–K**, respectively). Similar to previously mentioned results, *poxL* was required for nitrite-dependent inhibition (**Figures S6A–K** and **S7A–K**).

**Figure 5:**
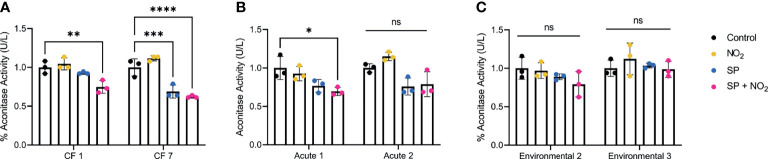
Clinical isolates have reduced aconitase activity in the presence of reactive nitrogen intermediates. **(A)** Clinical, **(B)** acute, and **(C)** environmental isolates of *P. aeruginosa* were grown in the presence of nitrite, *S. parasanguinis*, or nitrite and *S. parasanguinis.* Aconitase activity was recorded and normalized to the average of controls. n=3, error bars represent standard deviation, ns, P>0.05, *P<0.05, **P<0.01, ***P<0.001, ****P<0.0001 (Two-Way ANOVA, Tukey *post hoc* test).

Next, we wanted to determine if aconitase activity was impaired by *S. parasanguinis*-mediated RNI in chronic, acute, and environmental *P. aeruginosa* isolates as we observed in the FRD1 and PAO1 *norB* and *norC* mutant backgrounds. All CF isolates tested had reduced aconitase activity in the presence of *S. parasanguinis* and nitrite (**Figure 5**). Similar to the competition assays, acute isolates of *P. aeruginosa* had varied aconitase activity in the presence of RNI. Environmental isolates of *P. aeruginosa* did not have reduced aconitase activity in the presence of RNI, indicating that clinical *P. aeruginosa* isolates are distinctly more sensitive to RNI.

## Discussion

Our findings demonstrate that the oral commensal *S. parasanguinis* alters the global *P. aeruginosa* nitrosative stress response. Importantly, RNA sequencing revealed that the chronic CF isolate FRD1 had a reduced transcriptomic response to both nitrite and *S. parasanguinis*-mediated RNI compared to the acute isolate PAO1. Interestingly, the genes for nitric oxide reductase, *norCB*, were expressed at similar levels between the isolates, indicating that nitric oxide reductase is critical for *P. aeruginosa* tolerance to RNI. Moreover, we show that the nitric oxide reductase subunit B is essential to withstand nitrosative stress. Finally, we showed that enhanced sensitivity to *S. parasanguinis*-mediated RNI is not unique to only the chronic CF isolate FRD1, but to the majority of clinical CF isolates. Some clinical CF isolates were also sensitive to H_2_O_2_ production by *S. parasanguinis*, which suggests that chronic CF isolates also have reduced catalase activity since the growth of these isolates was restored when H_2_O_2_ production was abolished in *S. parasanguinis*. Interestingly, few environmental isolates were sensitive to *S. parasanguinis*-mediated RNI, indicating that there may be a regulatory switch in the nitrosative stress response as *P. aeruginosa* adapts to the host during chronic infections.

Previously, our lab has demonstrated that the nitrite reductase (*nirS*) is also required for *P. aeruginosa* to withstand *S. parasanguinis-*induced RNI and that FRD1 has reduced *nirS* transcription (Scoffield and Wu, 2016). While the accumulation of nitrite was deleterious in the *nirS* mutant, nitric oxide, the end product that accumulates when nitric oxide reductase is lost, is a much more reactive compound that influences *P. aeruginosa* signaling, behavior, and physiology (Mayburd and Kassner, 2002; Barraud et al., 2006; Ren et al., 2008; Barraud et al., 2009). Other groups have demonstrated that the accumulation or addition of nitric oxide can disperse biofilms and prevent *P. aeruginosa* colonization (Barraud et al., 2006; Barraud et al., 2009). Additionally, the absence of nitric oxide reductase makes *P. aeruginosa* more susceptible to killing by LPS-activated macrophages, further highlighting the necessity for *P. aeruginosa* to reduce nitric oxide during infection (Kakishima et al., 2007). Given the detrimental effects of nitric oxide on *P. aeruginosa*, clinical trials of NO-releasing nanoparticles have been explored for the treatment of *P. aeruginosa* (Schairer et al., 2012; Chatterjee et al., 2016).

Due to the cytotoxicity of nitric oxide, its conversion into less toxic compounds such as nitrous oxide and nitrogen are paramount for *P. aeruginosa* survival, especially in RNI-rich environments such as the CF lung and/or when in the presence of RNI-contributing organisms such as *S. parasanguinis* (Grasemann et al., 1998; Ho et al., 1998; Linnane et al., 1998; Scoffield and Wu, 2015). Our results indicate that while both the NorB and NorC subunits of nitric oxide reductase assist with the nitrosative stress response, NorB may be particularly critical for *P. aeruginosa* tolerance to RNI. The NorB subunit is the larger 53kDa subunit which is composed of 13 alpha helices that span the transmembrane space with one of these helices interacting directly with NorC (Hino et al., 2010; Yamagiwa et al., 2018). NorB contains two heme irons and one non-heme iron that act as the catalytic core of the enzyme. NorC is the smaller 17kDa membrane-anchored subunit which contains heme c that assists with electron entry into the complex. Not only is NorB known to assist with the conversion of nitric oxide into nitrous oxide, the subunit has also been shown to assist with the stabilization of the entire nitrate reduction complex (Borrero-de Acuña et al., 2016). The *norB* mutant has previously been found to have decreased nitrate and nitrite reduction abilities, suggesting that NorB is necessary for other denitrification enzymes to localize to the inner membrane or periplasm and/or function (Yoon et al., 2007; Borrero-de Acuña et al., 2016). Further, cell division proteins, metabolic enzymes involved in the TCA cycle and other metabolic pathways, and many protein transport proteins such as the Sec system were found to interact with the nitrate reduction megacomplex, which is anchored by NorCB (Borrero-de Acuña et al., 2016). Thus, our results highlight the importance and utility of NorB in the context of nitrosative stress and bacterial survival.

The research shown herein demonstrates that chronic CF *P. aeruginosa* isolates may be particularly sensitive to *S. parasanguinis-*mediated nitrosative stress. Increased sensitivity is likely due to deregulation of the denitrification process which has been observed in FRD1 and in various denitrification mutants of *P. aeruginosa* (Yoon et al., 2007; Scoffield and Wu, 2016). Regulation of the denitrification cascade is complex and involves multiple systems such as the master anaerobic nitrate regulators ANR and DNR, the NarXL nitrate-sensing two-component system, the flavohemoglobin regulator FhpR, and NirQ (Galimand et al., 1991; Arai et al., 1994; Arai et al., 1997; Arai et al., 2005; Schreiber et al., 2007). Further, when nitric oxide accumulates in bacteria, an iron sulfur cluster in ANR is nitrosylated, preventing binding to its cognate sequences leading to decreased transcription of nitrate and nitrite reductases (Yoon et al., 2007). Altered regulation of denitrification reductases by ANR along with our transcriptomic data, which suggests there is reduced denitrification gene expression in FRD1, supports our previous finding that FRD1 also has limited *nirS* expression (Scoffield and Wu, 2016).

While the denitrification complex is typically transcribed under the control of ANR in anaerobic conditions, aerobic denitrification has been shown to occur in microaerophilic conditions (Chen et al., 2003; Arat et al., 2015). Further, dioxygenases such as homogentisate-1,2-dioxygenase (HmgA) and 4-hydroxyphenylpyruvate dioxygenase (Hpd), which are primarily expressed under aerobic conditions, have been shown to be upregulated in the absence of *norCB* to scavenge nitric oxide (Yoon et al., 2007). Hence, the presence of NorCB may not only be utilized under canonical anaerobic denitrification but also in the bacteria’s response to nitrosative stress under microaerophilic conditions, as in our system. Additionally, chronic CF isolates of *P. aeruginosa* have been shown to undergo adaptations in metabolic, transcriptional, post transcriptional, and regulatory processes to adapt to the CF lung. These changes include but are not limited to alginate production, loss of motility, quorum sensing pathways, varied virulence factor production, and antibiotic resistance (Conrad et al., 1979; Pedersen et al., 1990; Hassett, 1996; Boucher et al., 1997; Shaver and Hauser, 2004; Palmer et al., 2005; Lindsey et al., 2008; Van Alst et al., 2009; Balasubramanian et al., 2013; Kafle et al., 2016; Winstanley et al., 2016; Pang et al., 2019). Many of these changes can directly or indirectly impact denitrification and tolerance to reactive nitrogen or oxygen species. For example, quorum sensing networks have been shown to be altered in clinical CF *P. aeruginosa* isolates, and deletion of RhlR or LasR leads to increased aerobic denitrification (Schaber et al., 2004; Toyofuku et al., 2007; Toyofuku et al., 2008; Feltner et al., 2016; Cui et al., 2021). Clinical CF isolates have also been shown to have reduced catalase due to AlgT negatively regulating *katA* transcription, which may explain the sensitivity of several CF isolates to *S. parasanguinis* alone (Malhotra et al., 2018). Thus, in addition to the contribution of *norCB*, there are likely additional varied factors that lead to increased RNI susceptibility by chronic CF *P. aeruginosa* isolates.

**Figure 6:**
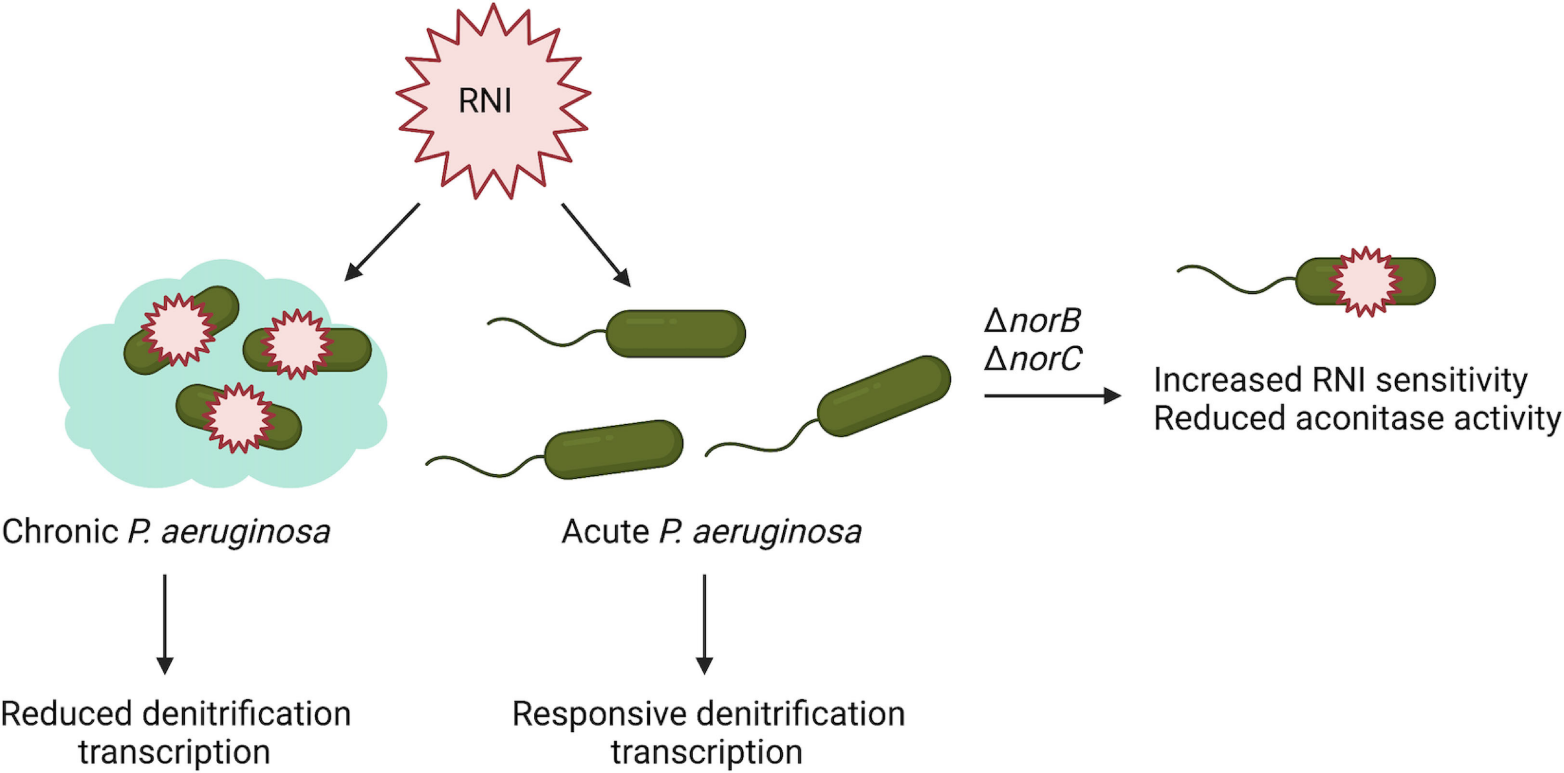
Proposed model showing altered denitrification expression renders chronic *P. aeruginosa* isolates more susceptible to commensal-mediated RNI. Chronic CF isolates of *P. aeruginosa* have increased sensitivity to RNI compared to acute or environmental isolates of *P. aeruginosa*, which may be in part due to altered expression of denitrification genes. Additionally, loss of *norB and norC* in an acute background recapitulates enhanced RNI sensitivity that is observed in chronic isolates.

In summary, our results demonstrate that 1) *S. parasanguinis* alters expression of genes that are critical for the *P. aeruginosa* nitrosative stress response, 2) nitric oxide reductase is essential for *P. aeruginosa* to withstand *S. parasanguinis-*mediated nitrosative stress, and 3) clinical CF isolates have increased susceptibility to *S. parasanguinis-*mediated RNI. While nitric oxide reductase likely contributes to RNI resistance both through reducing nitric oxide to nitrous oxide and through preventing nitric oxide accumulation that leads to loss of ANR function, there are likely other factors that contribute to increased RNI susceptibility that is observed in the CF isolates. Overall, our model illustrates that chronic CF isolates have lost the ability to elicit a robust protective response to RNI, and that nitric oxide reductase is critical for *P. aeruginosa* survival in the presence of RNI (**Figure 6**). Dysregulation of the denitrification pathway that occurs in the CF lung could potentially be exploited to decrease *P. aeruginosa* persistence and survival. Future work will determine the environmental queues and regulatory switches that occur in chronic *P. aeruginosa* infections that lead to increased susceptibility to RNI.

## Data Availability Statement

The datasets presented in this study can be found in online repositories. The names of the repository/repositories and accession number(s) can be found below: https://www.ncbi.nlm.nih.gov/, PRJNA779943.

## Author Contributions

JB and JS conceived and designed the study. JB performed plate inhibition, aconitase, and growth assays. JH prepared samples for RNA sequencing. SS performed aconitase assays. JS performed genetic complementation. Data analysis was performed by JB and JS. JB and JS drafted and edited the manuscript. All authors contributed to the article and approved the submitted version.

## Funding

This work was supported by grants awarded to JS from the National Institute of Dental and Craniofacial Research/NIDCR (R00DE025913) and the National Institute of General Medical Sciences (R35GM142748). This work was also funded by a UAB Cystic Fibrosis Research Center Pilot grant (P30DK072482) awarded to JS and start-up funds from the UAB Department of Microbiology. JB was supported by the NIDCR/Dental Academic Research Training Program (T90DE022736). SS was supported by the Alabama Louis Stokes for Minority for Participation fellowship funded by the National Science Foundation (1806130) and the National Heart, Lung, and Blood Institute (NHLBI) T32 UAB pre-doctoral training program in lung diseases (T32HL 134640-03), and is now supported by a NIH/NHLBI 1F31HL162487-01 National Research Service Award.

## Conflict of Interest

The authors declare that the research was conducted in the absence of any commercial or financial relationships that could be construed as a potential conflict of interest.

## Publisher’s Note

All claims expressed in this article are solely those of the authors and do not necessarily represent those of their affiliated organizations, or those of the publisher, the editors and the reviewers. Any product that may be evaluated in this article, or claim that may be made by its manufacturer, is not guaranteed or endorsed by the publisher.
