# Supplementary material for: A Commensal Streptococcus Dysregulates the Pseudomonas aeruginosa Nitrosative Stress Response
Source: Front Cell Infect Microbiol. 2022 May 10;12:817336. doi: 10.3389/fcimb.2022.817336 (PMC9127344; doi:10.3389/fcimb.2022.817336)
Supplement: Supplementary file 1 [file DataSheet_1.docx]

**
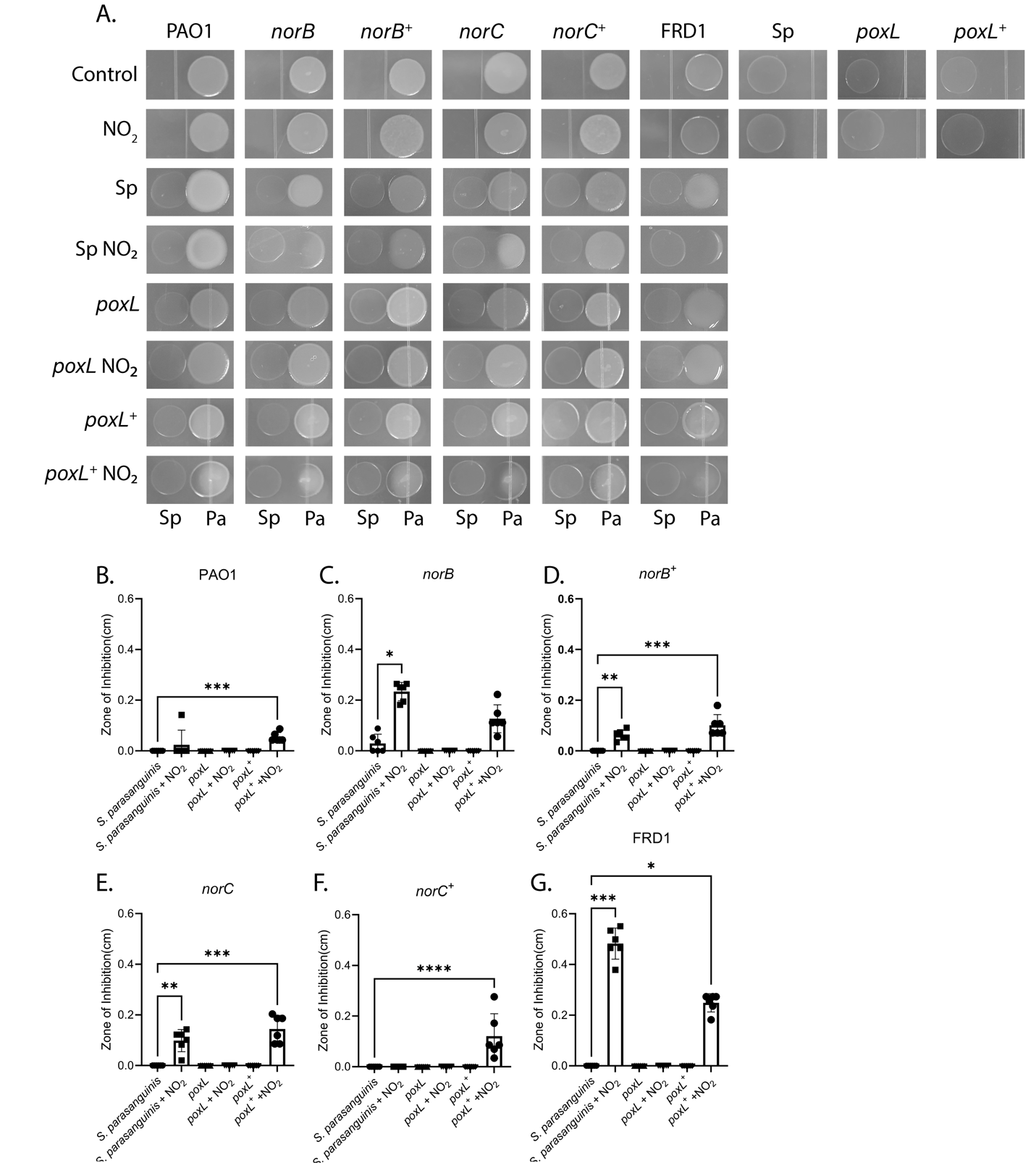
**

**Figure S1: *norB* and *norC* isolates are sensitive to *S. parasanguinis*-mediated RNI.**

A) Representative images of *S. parasanguinis* or *P. aeruginosa* spots with or without nitrite and competition assays between *S. parasanguinis, S. parasanguinis poxL, S. parasanguinis poxL^+^* and PAO1, *norB, norB^+^, norC, norC^+^,* and FRD1 in the presence or absence of nitrite. B-G) Zones of inhibition were quantified for each *P. aeruginosa* isolate and were compared by treatment. n=6, 3 biological replicates with 2 technical replicates, * P<0.05, *** P<0.001, **** P<0.0001 (Kruskal-Wallis, Dunnett Post Hoc Test).


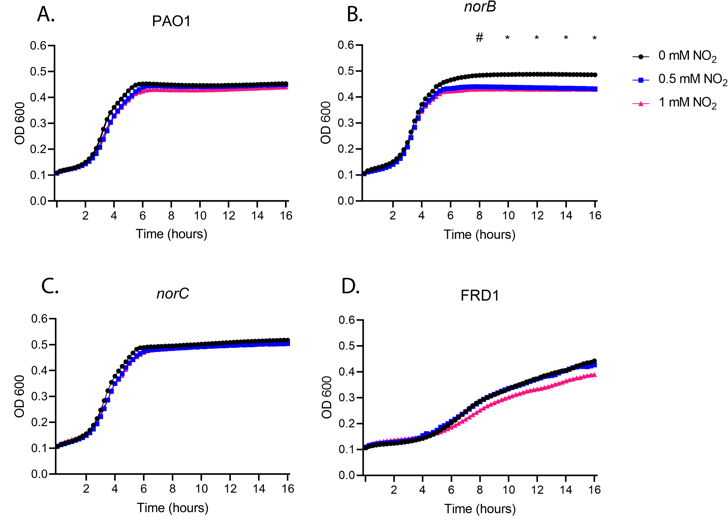


**Figure S2. FRD1 and *norB* have reduced growth in the presence of nitrite.**

16 hour growth curves of PAO1, FRD1, *norB, norC* in the presence of 0, 0.5, and 1mM nitrite. n=9, 3 biological replicates with 3 technical replicates, comparisons were made at each timepoint, #P <0.05 0mM NO_2_ vs 1mM NO_2_, * P<0.05 0.5mM, 1mM NO_2_ versus 0mM (Two-Way ANOVA, Dunnett Post Hoc Test).


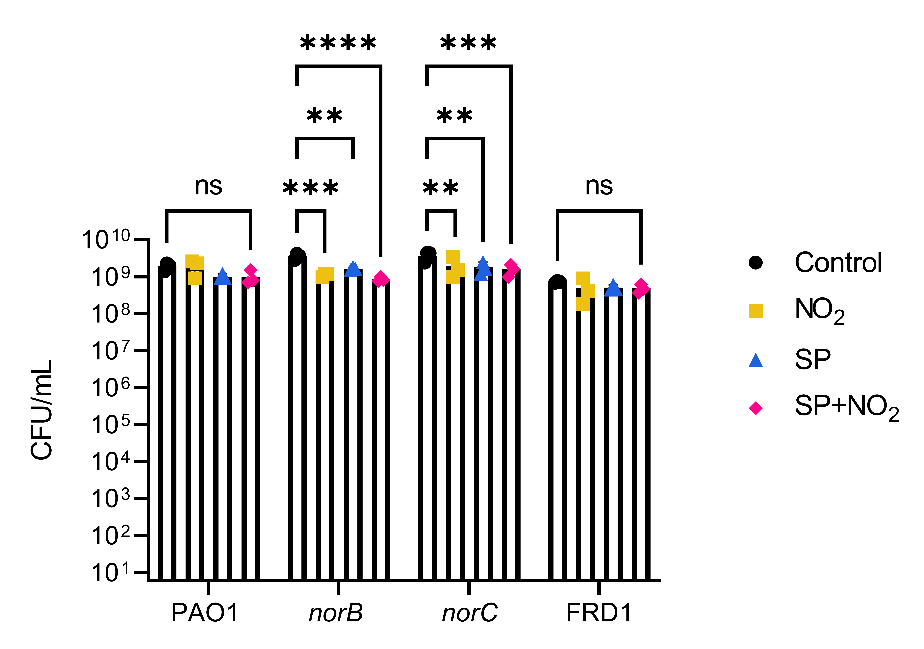


**Figure S3. Transwell co-culture of *S. parasanguinis* and *P. aeruginosa* does not kill PAO1 or FRD1*.***

After the five hour incubation with *S. parasanguinis* grown in the insert in the presence or absence of nitrite, *P. aeruginosa* CFU were quantified through serial dilution and spot plating. PAO1 and FRD1 CFU were not significantly reduced from controls n=3, ** P<0.01, *** P<0.001, **** P<0.0001 (Two-Way ANOVA, Tukey Post Hoc Test).


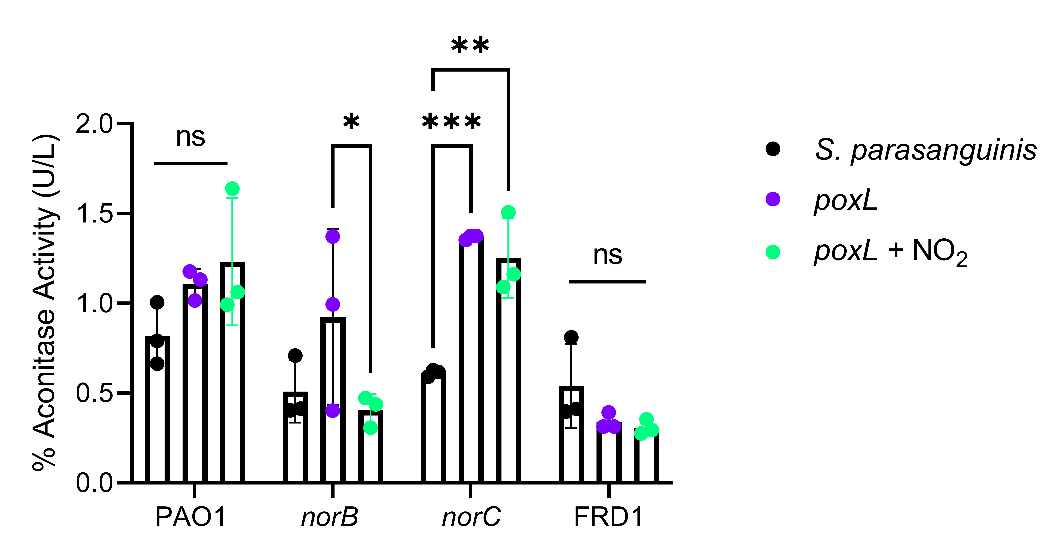


**Figure S4. Nitrite or RNIs are required for *norB* sensitivity*.***

PAO1, *norB, norC,* and FRD1 were exposed to *S. parasanguinis*, *S. parasanguinis poxL,* and *S. parasanguinis poxL* and nitrite*.* Aconitase activity was measured and normalized to the average of the controls. n=3, * P<0.05, ** P<0.01, *** P<0.001, (Two-Way ANOVA, Tukey Post Hoc Test).


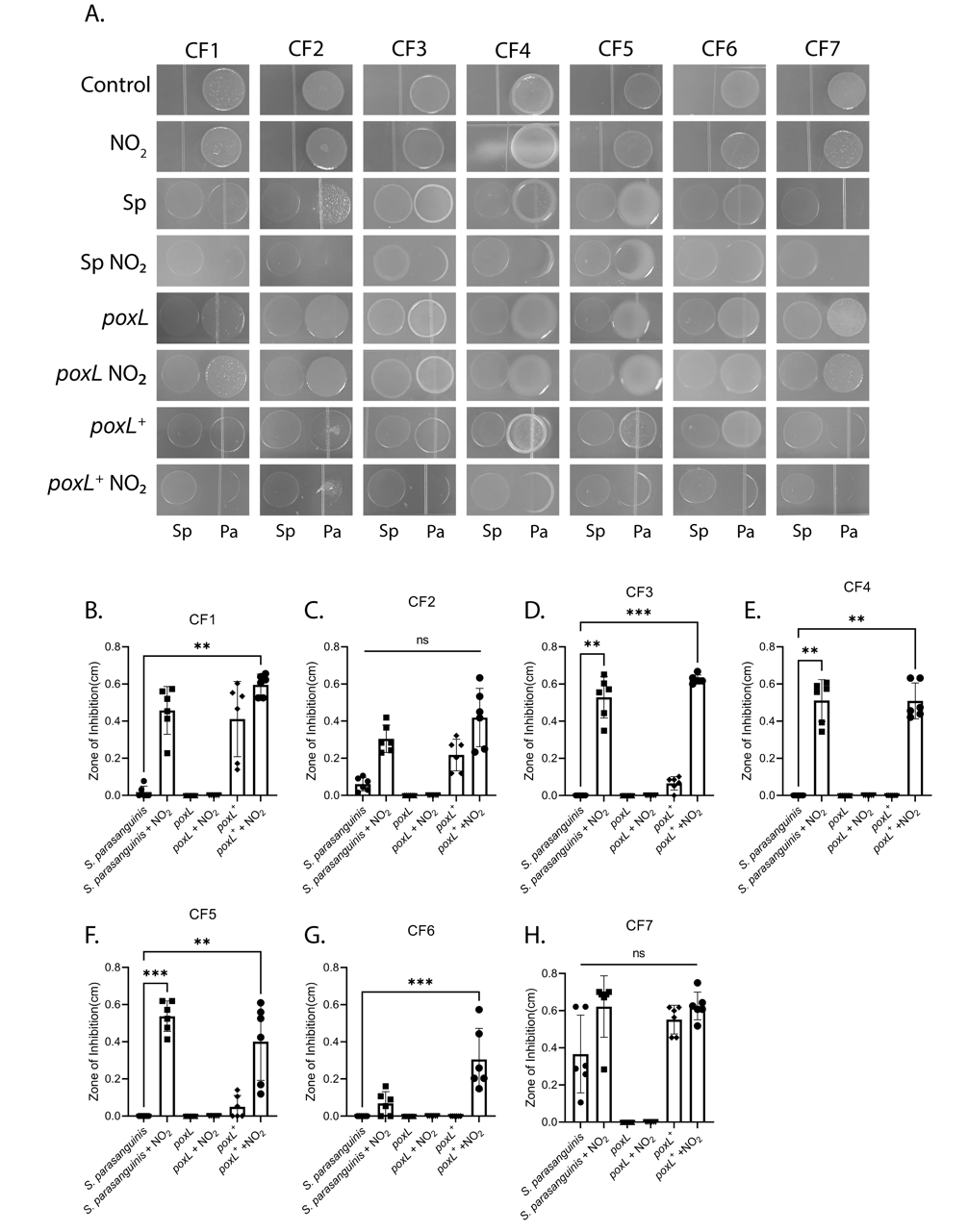


**S5. CF isolates of *P. aeruginosa* are especially sensitive to *S. parasanguinis-*induced nitrosative stress.**

A) CF isolates of *P. aeruginosa* were spotted next to *S. parasanguinis, S. parasanguinis poxL,* and *S. parasanguinis poxL+* in the presence or absence of 1mM nitrite. B-H) Zones of inhibition were quantified for each isolate and were compared by treatment. n=6, 3 biological replicates with 2 technical replicates, ** P<0.01, *** P<0.001, (Kruskal-Wallis, Dunnett Post Hoc Test).


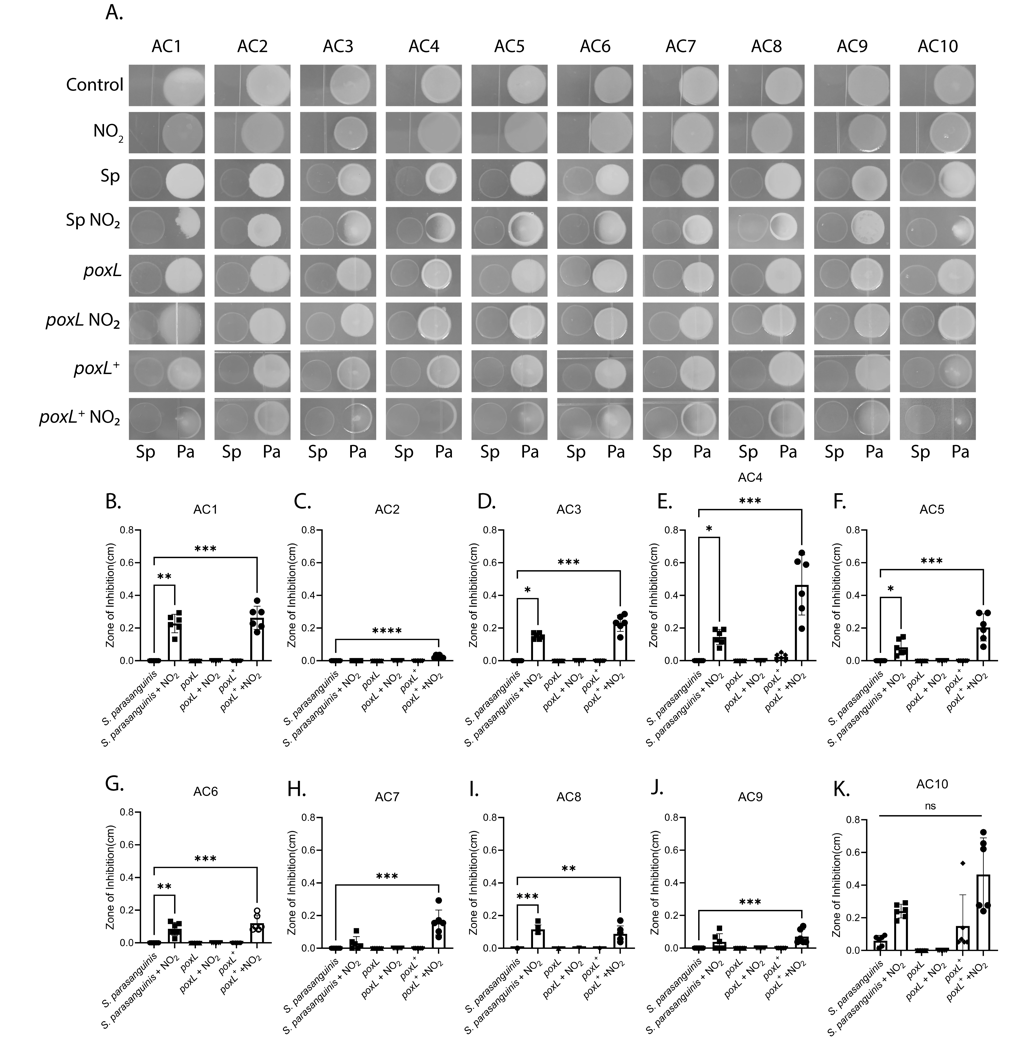


**S6. Acute isolates have varied sensitivity to RNI.**

A) Acute isolates of *P. aeruginosa* were spotted next to *S. parasanguinis, S. parasanguinis poxL,* and *S. parasanguinis poxL+* in the presence or absence of 1mM nitrite. B-K) Zones of inhibition were quantified for each isolate and were compared by treatment. n=6, 3 biological replicates with 2 technical replicates, ** P<0.01, *** P<0.001, **** P<0.0001 (Kruskal-Wallis, Dunnett Post Hoc Test).


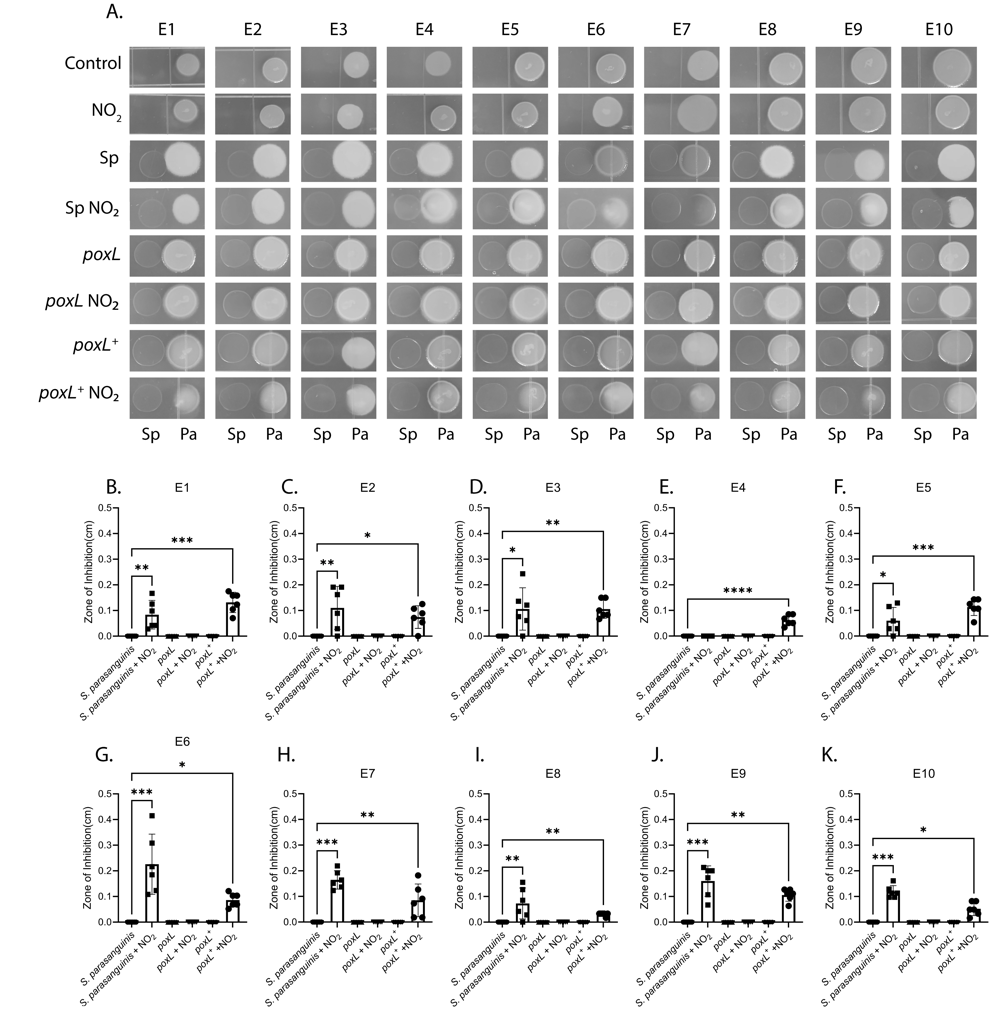


**S7. Environmental isolates have reduced sensitivity to RNI.**

A) Environmental isolates of *P. aeruginosa* were spotted next to *S. parasanguinis, S. parasanguinis poxL,* and *S. parasanguinis poxL^+^* in the presence or absence of 1mM nitrite. B-K) Zones of inhibition were quantified for each isolate and were compared by treatment. n=6, 3 biological replicates with 2 technical replicates, * P<0.05, ** P<0.01, *** P<0.001, **** P<0.0001 (Kruskal-Wallis, Dunnett Post Hoc Test).
